# The diverse metabolic heterogeneity of stem cells in a BRCA+/-breast cancer population

**DOI:** 10.25122/jml-2020-0105

**Published:** 2021

**Authors:** Svetlana Mykolaivna Gramatiuk, Irina Yuriivna Bagmut, Michael Ivanovich Sheremet, Vitaliy Vasilyevich Maksymyuk, Volodimir Volodimirovich Tarabanchuk, Petro Vasilyevich Moroz

**Affiliations:** 1. Institute of Cellular Biorehabilitation, Ukraine Association of Biobank Kharkiv, Ukraine; 2. Kharkiv Medical Academy of Postgraduate Education, Kharkiv, Ukraine; 3. Surgical Department No. 1, Bukovinian State Medical University, Chernivtsi, Ukraine

**Keywords:** stem-cell behavior, mitochondria, BRCA1 gene, MCF-7 breast cancer cells, breast cancers

## Abstract

Breast cancers are very heterogeneous tissues constituted by epithelial cancer cells and an abnormal tumor microenvironment – cancer-associated fibroblasts (CAFs), activated adipocytes, mesenchymal stem cells (MSCs), and others. The aim of the study is to cancer cells and their microenvironment, which behave like a complex and heterogeneous metabolic ecosystem, where cancer cells can reprogram their metabolism as a result of interaction with the components of the microenvironment. The study was based on cancer stem cells (CSC) that were isolated from breast tumors by magnetic separation (AutoMACS). We used spectrophotometric methods for the measurement of aldehyde dehydrogenase (ALDH) enzymatic activity. For these experiments, we used breast cancer and normal stem cell lines. Analyses showed that the proportion of BRCA+ CSC cells was in accordance with the relatively low percentages of CSCs in BRCA+ tumors. ALHD was significantly higher in the CSCs-high BRCA+ breast cancer and CSCs-low BRCA- breast cancer cells, compared with the CSCs-low BRCA+ breast cancer. Breast cancer from BRCA mutation carriers harbor more “high-energy” cell sub-populations than “low-energy” and have their more aggressive phenotype. Key oncogenic pathways known to be dysregulated in breast cancer also regulate stem-cell behavior.

## INTRODUCTION

In each cellular process stage, from signal transduction of cellular metabolism to cell death, mitochondria play a key role. Taking into account that these features are essential for cancer growth, the application point for anti-cancer therapy can be focused on the mitochondria. It should also be mentioned that mitochondria participate not only in the regulation of stem cell identity and differentiation but also in outcomes. The critical role of mitochondria has also been shown in malignant stem-like cells termed cancer stem cells (CSCs), which are involved in the progression and resistance of different kinds of tumors [1–4].

Breast cancers belong to diverse tissues represented by epithelial cancer cells and an abnormal tumor microenvironment including blood and lymphatic tumor vessels, an extracellular matrix (ECM), and non-cancer stromal cells constituted by endothelial and immune cells, pericytes and mesenchymal stem cells (MSCs), activated adipocytes and cancer-associated fibroblasts (CAFs).

However, cancer cells represent a tissue with a similar complex and heterogeneous metabolic ecosystem with the ability to reprogram their metabolism as a result of interaction with microenvironment factors [5–8]. Generally, breast cancer cells can be divided into three groups: “bulk” cancer cells, which represent 85–95% of the population, progenitor cells (5%), and cancer stem cells (CSCs) (1%). The most dangerous of them are progenitor cells and CSCs because they can act as tumor-initiating cells (TICs) in vivo and are responsible for metastasis. On the contrary, “bulk” cancer cells belong to a cell population with low tumorigenic potential.

Heterozygous germline mutations in the BRCA1 gene predispose women to up to an 80% lifetime risk of developing breast cancer [6, 9–14]. Most of these tumors have the basal phenotype, characterized by the expression of myoepithelial markers, but lack the expression of ER, PR, and ERBB2 receptors.

Nevertheless, one of the fantastic properties of breast cancer cells continues to be their metabolic plasticity. Notably, the phenomenon known as the Warburg effect relates to a shift from mitochondrial oxidative phosphorylation (OXPHOS) to glycolysis in the presence of oxygen in breast cancer cells which is the most critical process for energy-saving and rapid proliferation [15–19].

### Clinical implications of the cancer stem-cell hypothesis

The cancer stem-cell hypothesis has fundamental implications for breast carcinogenesis as well as important clinical implications for prevention and therapy. In our research, we wanted to test the following hypothesis:

There are heterogeneous metabolic types in the CSC breast cancer BRCA+/- population;

Clinical, molecular, and pathologic features of breast cancer in BRCA1 mutation carriers suggest the possibility that BRCA1 may function as a stem-cell regulator.

### Study details

In the first step, stem cells were isolated from the breast cancer BRCA+/- population. In the second step, stem cells from conditionally healthy donors from the Ukrainian Association of Biobank under the CIMBA project were obtained. Lastly, the third step included laboratory in vivo research on the clinical, molecular, and pathologic features CSCs breast cancer in BRCA1 and BRCA2 mutations. Multiple analyses were conducted for CSCs, including the breast cancer BRCA+/- population phenotype, aldehyde dehydrogenase expression, mammosphere formation, and the magnetic-activated cell sorting approach to metabolically fractionate the breast cancer cell population into “low-energy” and “high-energy” cell sub-populations.

## MATERIAL AND METHODS

Isolation of cancer stem cells from primary human BRCA+ and BRCA-breast tumors using magnetic-activated cell sorting (MACS) from patients with breast cancer was performed in the context of a clinical protocol. CSCs were isolated from breast tumors using the autoMACS technology. This was followed by seeding 50,000 mononucleated cells/cm^2^ in an RPMI (1x) + GlutaMAX medium (Gibco Life Technologies, Canada) supplemented with 10% fetal bovine serum (Thermo Fisher Scientific, USA) in a Tissue Culture Dish (BioLite 60mm, Thermo Fisher Scientific, USA) [20].

The cultures were incubated at 37°C, 20% O_2_, 5% CO_2_ using the automatic Fibra Stage system (New Brunswick Scientific, USA). Medium changes were performed twice a week. Two weeks after the initial seeding, primary CSCs colonies were separated after 10 mini-incubations at 37°C with Trypsin-EDTA 0.05% (Gibco Life Technologies, Canada) and replaced with 4000 cells/cm^2^ in the same medium. Subsequent passages were performed following the same steps. Passages 4–6 were used for all experiments [21].

MACS can be used to select metabolically “low-energy” and “high-energy” cell subpopulations of breast cancer and test this hypothesis [6]. Their energetic state can be analyzed using auto-fluorescence assigned to the endogenous flavin-containing metabolites: flavin mononucleotide (FMN), flavin adenine dinucleotide (FAD) and riboflavin.

We used spectrophotometric methods to measure the effects of siRNA expression, particularly the enzymatic activity of ALDH.

First, from each of the stem cell lines (cancer and normal cells), the lysates were separated according to size in parallel on two 12% denaturing SDS-polyacrylamide gels (BioRad, Hercules, CA). Afterward, they were electrotransferred onto nitrocellulose membranes and blocked with 5% milk in tris-buffered saline (TBS). A substrate of cell lysate, 5 mM NAD+, 5 mM propionaldehyde and 600 μl lysing buffer was incubated using spectrophotometer cuvettes (Dynex Technology, USA) at 37°C.

In three replicates, the rate of change in absorbance at 340 nm was assessed over 5 min. The endogenous rate of NAD+ reduction was also monitored in the control reaction without a substrate. The ALDH activity was measured in nmoles/10 cells min [21]. The cells were collected and automatically counted after 72 hours of growth in 6-well tissue culture plates of triplicates of 2×10 cells/ml/well. The breast cancer and normal stem cell lines were used. Triplicates of 200 cells/ml/well for each cell line were cultured in a six-well plate in an RPMI-1640 medium with 10% Fetal Bovine Serum (FBS). Growth time was 5 days.

The number of colonies adhered to the bottom of the plate was counted using an inverted microscope. The in vitro scratch wound migration assay was used to count the downregulation of ALDH on cell migration and motility process. From five different cell lines, 23 monolayer confluent cells were received and scratched with the sterile pipette tip. The migration ability of the cells was measured with a digital camera after 20 hours in an RPMI-1640 medium +10% FBS. The width of the wound was examined under an inverted microscope.

Scratch wound images were taken straight after the scratch and 20 hours later. The migration distances (in cm) of the cells were measured on printed images taken at the same magnification. The migration distances of the cells and the width of wounds at the beginning and 20 hours later were measured, and the wound healing percentage was evaluated.

### Statistical analysis

Tests were used for the three independent experiments. The results were expressed as mean and standard deviation. Analysis of variance (one-way ANOVA) was performed in combination with Dunnett’s post-test for multiple comparisons. Correlations between STAT1 and PLSCR1 were determined by Pearson’s correlation and Spearman’s rank correlation test. Differences from the controls were considered significant as *p-0.05, **p-0.001 and***p-0.0001. The GraphPad Prism software (version 5.03) was used for all statistical assessments.

## RESULTS AND DISCUSSION

**Figure 1:**
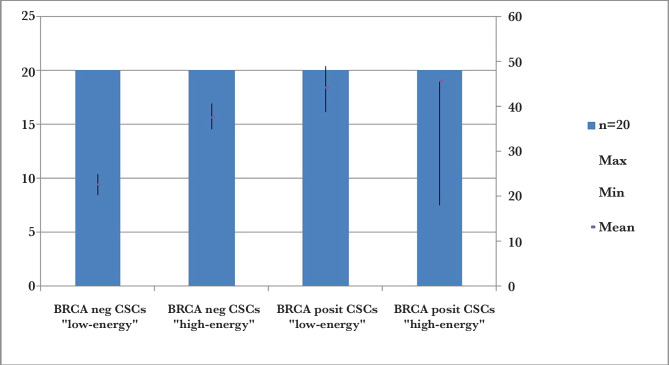
The magnetic separation system approach to metabolically fractionate the BRCA-/+ breast cancer cell population into “low-energy” and “high-energy” cell subpopulations.

Isolation of cancer stem cells from primary human BRCA+ and BRCA-breast tumors using MACS were collected in the context of a clinical protocol. To test this hypothesis, we used the magnetic separation approach to metabolically fractionate the breast cancer cell population into “low-energy” and “high-energy” cell subpopulations. For this purpose, we used auto-fluorescence as an endogenous marker of their energetic state riboflavin (vitamin B2). Using vitamin B2 as a fluorescent indicator, we found that BRCA+ CSCs showed a significant reduction in the population of breast cancer cells to “high-energy”, and BRCA- CSCs reduced the number of breast cancer cells in the population to “low-energy” (Figure 1).

Thus, BRCA + CSCs reduced the level of “high-energy” population of breast cancer cells to 25% (n=20; p=0.001); the population of “low-energy” BRCA- CSCs was 56% (n=20; p=0.001).

**Figure 2:**
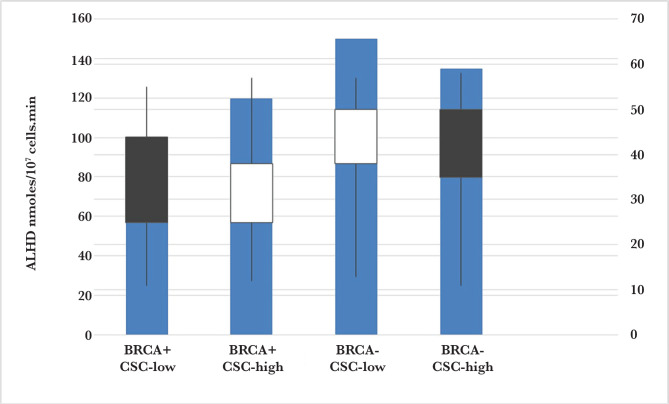
ALDH assay and cell sorting of cancer stem cells from primary human BRCA+ and BRCA- breast tumors.

ALDH assay and cell sorting were performed in cancer stem cells from primary human BRCA+ and BRCA- breast tumors. The results of the spectrophotometer cuvettes were noted after the addition of cell lysate, 5 mM NAD+ and 5 mM propionaldehyde as a substrate. The rate of change in absorbance at 340 nm was measured in 3 replicates over 5 min analyses and showed that the proportion of BRCA+ CSC cells was 35.12±0.63 nmoles/107 cells per min (Figure 2), which was in accordance with the relatively low percentages of CSCs in BRCA+ tumors. ALHD was significantly higher in the CSCs-high BRCA+ breast cancer (45.1±2.3%) and CSCs-low BRCA-breast cancer (33.6±1.4%) cells, compared with the CSCs-low BRCA+ breast cancer (1.7±0.53%) cells.

Loss of BRCA functions results in the genomic instability that eventually results in the oncogenic transformation of non-tumorigenic cells into tumor-initiating cells or cancer stem cells and further tumor evolution. A CSC population possesses a long-term self-renewal capacity, as well as a potential to differentiate into other tumor cell types and initiate tumor growth. As essential regulators of genome stability and gene expression, BRCA1/2 plays an important role in CSCs development and evolution and can be employed in different CSC signaling mechanisms [25–27].

ALDH can be present in the cytosol, the endoplasmic reticulum, mitochondria and even the nucleus, sometimes in more than one place. ALDH is also located in stem cells. During the first stage of life and growth, stem cells have a strong potential to repair the internal system.

They can develop into different cell types in many tissues and divide to restore others without limit [3].

High ALDH activity has been noted in liver cells or the bronchial epithelium of smokers caused by carcinogenic aldehydes in cigarette smoke. Ginestier *et al*. stated that ALDH1 is a marker for stem/progenitor cells of the normal breast. It is also active in stem cells of several tumors like breast cancer or lung cancer. Its activity might be crucial for both stem cell longevity and the resistance of CSCs to chemotherapy [28–31].

ALDH expression has demonstrated itself to be a possibly relevant prognostic marker. For this reason, the subpopulation of cancer SCs can be a therapeutic target in cases with a poor prognosis, treatment-resistant and recurrent breast cancer. At the same time, the ALDH+ population isolated from breast tumor specimens displayed a high tumorigenic potential in mice models [32–35]. BRCA1 plays a role in the self-renewal of progenitor cells, a fact which was demonstrated by serial passaging of BRCA1- deficient normal mammary epithelial cells where the number of mammospheres increased with the number of passages [36–38].

## CONCLUSION

Accumulating evidence suggests that CSCs exist as a subpopulation of quiescent cells within the dominant tumor bulk of heterogeneous tumor cells. The research on self-renewal pathways that regulate the self-renewal of breast stem cells has led to a clearer picture of how dysregulation of these pathways may lead to carcinogenesis. Furthermore, these pathways may provide targets for breast cancer prevention and therapy. Indeed, recent evidence suggests that key oncogenic pathways known to be dysregulated in breast cancer also regulate stem-cell behavior. Breast cancer from BRCA mutation carriers harbor more “high-energy” cell subpopulations than “low-energy”,and have a more aggressive phenotype.
